# TEMPORARY ADDITIONS TO TEMPORARY HOUSING: LESSONS FROM HEALTH EXPERIENCES OF COVID AVOIDANCE HOTEL RESIDENTS

**DOI:** 10.1093/geroni/igac059.1331

**Published:** 2022-12-20

**Authors:** Ian Johnson, Michael Light, Tam Perry, Terri Lewinson

**Affiliations:** University of Tennessee, Knoxville, Tennessee, United States; University of Washington Palliative Care Training Center, Seattle, Washington, United States; Wayne State University, Detroit, Michigan, United States; The Dartmouth Institute for Health Policy and Clinical Practice, Lebanon, New Hampshire, United States

## Abstract

COVID-19 amplified system burdens and health risks within the housing care continuum, in which older adults with chronic serious illness are disproportionately represented. We present retrospective chart review data about the health experiences of older adults with serious illness living in and moving through temporary avoidance hotels during the COVID-19 pandemic. Through narratives of fourteen residents, we illustrate trends across two nine-month phases. Trends illustrate how avoidance hotels created opportunities for continuity of care, connection to services, and health-affirming relationships with place. We also identified challenges in catering to diverse medical, behavioral, and psychosocial-spiritual needs of older and seriously ill residents, as well as negative consequences to the geographic dispersion caused by de-congregating homeless shelters. Avoidance hotels present important lessons in considering future housing and healthcare intervention and implementation for older people facing homelessness while seriously ill.

